# Association of brain tissue cerebrospinal fluid fraction with age in healthy cognitively normal adults

**DOI:** 10.3389/fnagi.2023.1162001

**Published:** 2023-06-16

**Authors:** Liangdong Zhou, Yi Li, Elizabeth M. Sweeney, Xiuyuan H. Wang, Amy Kuceyeski, Gloria C. Chiang, Jana Ivanidze, Yi Wang, Susan A. Gauthier, Mony J. de Leon, Thanh D. Nguyen

**Affiliations:** ^1^Department of Radiology, Weill Cornell Medicine, New York, NY, United States; ^2^Penn Statistics in Imaging and Visualization Endeavor (PennSIVE), Department of Biostatistics and Epidemiology, University of Pennsylvania, Philadelphia, PA, United States; ^3^Department of Statistics and Data Science, Cornell University, Ithaca, NY, United States; ^4^Meinig School of Biomedical Engineering, Cornell University, Ithaca, NY, United States

**Keywords:** T2 relaxometry, cerebrospinal fluid fraction (CSFF), myelin water fraction (MWF), intra/extra-cellular water fraction (IEWF), FAST-T2, normal aging, perivascular space

## Abstract

**Background and purpose:**

Our objective was to apply multi-compartment T2 relaxometry in cognitively normal individuals aged 20–80 years to study the effect of aging on the parenchymal CSF fraction (CSFF), a potential measure of the subvoxel CSF space.

**Materials and methods:**

A total of 60 volunteers (age range, 22–80 years) were enrolled. Voxel-wise maps of short-T2 myelin water fraction (MWF), intermediate-T2 intra/extra-cellular water fraction (IEWF), and long-T2 CSFF were obtained using fast acquisition with spiral trajectory and adiabatic T2prep (FAST-T2) sequence and three-pool non-linear least squares fitting. Multiple linear regression analyses were performed to study the association between age and regional MWF, IEWF, and CSFF measurements, adjusting for sex and region of interest (ROI) volume. ROIs include the cerebral white matter (WM), cerebral cortex, and subcortical deep gray matter (GM). In each model, a quadratic term for age was tested using an ANOVA test. A Spearman’s correlation between the normalized lateral ventricle volume, a measure of organ-level CSF space, and the regional CSFF, a measure of tissue-level CSF space, was computed.

**Results:**

Regression analyses showed that there was a statistically significant quadratic relationship with age for CSFF in the cortex (*p* = 0.018), MWF in the cerebral WM (*p* = 0.033), deep GM (*p* = 0.017) and cortex (*p* = 0.029); and IEWF in the deep GM (*p* = 0.033). There was a statistically highly significant positive linear relationship between age and regional CSFF in the cerebral WM (*p* < 0.001) and deep GM (*p* < 0.001). In addition, there was a statistically significant negative linear association between IEWF and age in the cerebral WM (*p* = 0.017) and cortex (*p* < 0.001). In the univariate correlation analysis, the normalized lateral ventricle volume correlated with the regional CSFF measurement in the cerebral WM (ρ = 0.64, *p* < 0.001), cortex (ρ = 0.62, *p* < 0.001), and deep GM (ρ = 0.66, *p* < 0.001).

**Conclusion:**

Our cross-sectional data demonstrate that brain tissue water in different compartments shows complex age-dependent patterns. Parenchymal CSFF, a measure of subvoxel CSF-like water in the brain tissue, is quadratically associated with age in the cerebral cortex and linearly associated with age in the cerebral deep GM and WM.

## 1. Introduction

The cerebrospinal fluid (CSF) system plays a prominent role in maintaining the homeostasis of the central nervous system, providing hydromechanical protection, nutrient transport, and metabolic waste removal, among other essential functions (Sakka et al., 2011). In the brain, CSF is present within the ventricles, the cranial subarachnoid space, and the perivascular space (PVS). The PVS surrounds blood vessels and acts as a conduit for the exchange of fluid between CSF and the interstitial space (Wardlaw et al., 2020). Advanced imaging techniques have shown that the PVS is a key component of the glymphatic system for brain waste removal (Weller et al., 1992; Iliff et al., 2012; Nedergaard, 2013; Taoka et al., 2017). PVS dilation occurs during normal aging (Kim et al., 2022) and has been established as an early imaging marker of cerebral small vessel disease (Wardlaw et al., 2013), cerebral amyloid angiopathy (van Veluw et al., 2016; Charidimou et al., 2017), and Alzheimer’s disease (Banerjee et al., 2017; Boespflug et al., 2018). Traditionally, visible PVS enlargement on T2-weighted (T2W) images is assessed by counting or segmenting hyperintense punctate foci in the white matter (WM) centrum semiovale and the basal ganglia (Potter et al., 2015; Ramirez et al., 2016; Ballerini et al., 2018). However, this method cannot reliably detect PVS smaller than 100 micrometers, which is much smaller than the typical 1 mm imaging resolution.

Multi-component T2 relaxometry is a quantitative MRI method that can separate signals from different water compartments within an imaging voxel based on their T2 relaxation times (Whittall et al., 1997). In healthy brain gray matter (GM) and WM tissues, three water compartments can be distinguished based on the degree of water mobility, including water trapped in the myelin sheath with short T2 (∼10 ms), intra/extra-cellular water with intermediate T2 (∼70 ms), and CSF with long T2 (∼2 s) (MacKay et al., 2006). Of these, myelin water fraction (MWF), expressed as the ratio between the myelin water volume and the total water volume in a voxel (MacKay et al., 1994), has been established as an imaging marker of myelin injury in multiple sclerosis (MS) and other demyelinating disorders (Laule et al., 2008; Mancini et al., 2020; van der Weijden et al., 2021). This has spurred the development of a number of fast MRI techniques for MWF mapping (Alonso-Ortiz et al., 2015), including 3D gradient echo spin echo (GRASE) (Prasloski et al., 2012), 3D multi-component driven equilibrium steady-state observation of T1 and T2 (mcDESPOT) (Deoni et al., 2008), and 3D fast acquisition with spiral trajectory and adiabatic T2prep (FAST-T2) (Nguyen et al., 2016) sequences. To date, however, there have been few MRI studies on the parenchymal CSF fraction (CSFF) in normal aging, which could serve as a quantitative biomarker of PVS dilation on the microscopic scale. In a recent study, Canales-Rodriguez et al. (2021) applied multi-component T2 relaxometry to the GRASE brain MRI data of cognitively normal (CN) subjects, and reported a linear increase of CSFF with age. However, their imaging voxel size was rather large (42.9 mm^3^) and they did not include subjects aged 60 years or above. Therefore, the objective of this work was to demonstrate the feasibility of the FAST-T2 sequence with a smaller voxel size (7.8 mm^3^) for CSFF mapping and to study the effect of aging on parenchymal CSFF in CN individuals aged 20–80 years.

## 2. Materials and methods

### 2.1. Study participants

This was a cross-sectional study conducted in a cohort of 60 CN volunteers {24 men (40.0%), 36 women (60.0%); mean age, 49.6 years ±17.4 [standard deviation (SD)]; age range, 22.4–79.8 years} who had brain MRIs with the FAST-T2 sequence performed as part of imaging research studies at Weill Cornell Medicine. The mean age was 50.3 years ±16.7 (range, 23.2–74.2 years) for women and 48.6 ± 19.1 (range, 22.4–79.8 years) for men. All studies were approved by the local institutional review board and written informed consent was obtained from all participants prior to imaging. Subjects contraindicated for MRI, or those with a history of neurological problems (including, but not limited to, stroke, head trauma, brain infarcts, intracranial mass, MS, meningitis, encephalitis, prior brain surgery), or those on medications that may affect cognitive performance, were excluded. To rule out older subjects with potential cognitive deficits, 27 out of 30 subjects aged 50 years or more also underwent extensive neurocognitive assessments which included the Brief Cognitive Rating Scale (Reisberg and Ferris, 1988), the Global Deterioration Scale (GDS) (Reisberg et al., 1982), and the Clinical Dementia Rating (CDR) (Morris, 1993).

### 2.2. MRI examination

All 60 subjects underwent brain MRIs on a Siemens Prisma 3T scanner (Siemens Healthineers, Erlangen, Germany) using a product 64-channel head/neck receiver coil. The brain imaging protocol consisted of 3D T1-weighted (T1W) magnetization-prepared rapid acquisition gradient echo (MPRAGE) and 2D T2W turbo spin echo (TSE) or 3D T2W Sampling Perfection with Application optimized Contrast using different flip angle Evolution (SPACE) sequences for anatomical structure, as well as 3D FAST-T2 sequence for water fraction (WF) mapping. Study participants aged 45 years or above also had a 3D fluid-attenuated inversion recovery (FLAIR) SPACE scan for the detection of WM hyperintensities (WMH). The amplitude, shape, and timing parameters of the radiofrequency and gradient pulses in the FAST-T2 sequence were the same for all participants. The imaging parameters were as follows: (1) 3D sagittal MPRAGE: TR/TE/TI = 2,300/2.3/900 ms, flip angle (FA) = 8^°^, readout bandwidth (rBW) = 200 Hz/pixel, voxel size = 1.0 mm isotropic, GRAPPA parallel imaging factor (R) = 2, scan time = 5.5 min, (2a) 2D axial T2W TSE: TR/TE = 5,840/93 ms, FA = 90^°^, rBW = 223 Hz/pixel, turbo factor = 18, number of signal averages (NSA) = 2, voxel size = 0.5 × 0.8 × 3.0 mm^3^, R = 2, scan time = 4 min; or alternatively, (2b) 3D sagittal T2W SPACE: TR/TE = 3,200/408 ms, FA = 90^°^, rBW = 751 Hz/pixel, turbo factor = 285, voxel size = 1.0 mm isotropic; (3) 3D axial FAST-T2 (Nguyen et al., 2016): spiral TR/TE = 7.8/0.5 ms, nominal T2prep times = 0 (T2-prep turned off), 7.5, 17.5, 67.5, 147.5, and 307.5 ms, T1 saturation recovery time (wait time between the saturation pulse at the end of spiral readout and the next T2prep) = 2 s, FA = 10^°^, rBW = 1,042 Hz/pixel, number of spiral leaves per stack = 32, number of spiral leaves collected per T2prep = 64, voxel size = 1.3 × 1.3 ×5 mm^3^, scan time = 4 min; (4) 3D sagittal FLAIR SPACE with fat saturation: TR/TE/TI = 4,000/384/2,400 ms, echo spacing = 3.46 ms, FA = 90^°^, rBW = 751 Hz/pixel, turbo factor = 278, voxel size = 1.0 mm isotropic, R = 2, scan time = 5.4 min.

To evaluate the intra-scanner repeatability of the FAST-T2 sequence, seven subjects (five women, two men; mean age, 28.4 years ±4.4; age range, 22.4–33.6 years) also underwent a second FAST-T2 scan in a separate scanning session on the same day (subjects exited the scanner and were repositioned between sessions).

### 2.3. Image post-processing

**FIGURE 1:**
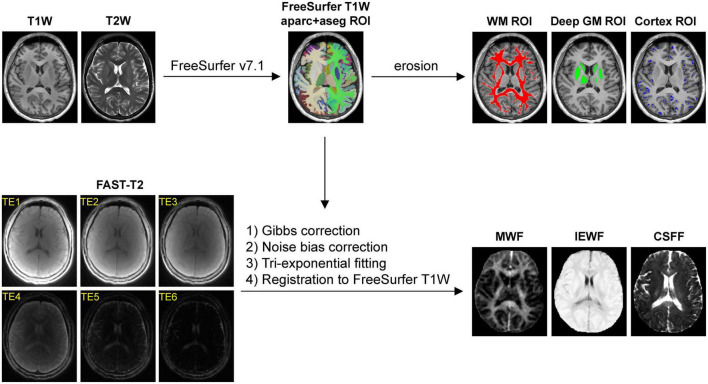
Diagram of the automated image post-processing pipeline, consisting of (1) brain segmentation and cortical parcellation of the T1-weighted (T1W) images by FreeSurfer, followed by an erosion operation (1 mm in-plane and 5 mm through-plane) to generate the cerebral white matter (WM), gray matter (GM), and cortical masks for region of interest (ROI) analysis, and (2) extraction of the water fraction maps from the Gibbs- and noise-corrected six-echo fast acquisition with spiral trajectory and adiabatic T2prep (FAST-T2) data, followed by their registration to the FreeSurfer T1W space.

Figure 1 summarizes the main steps of the automated image post-processing pipeline. Regional brain segmentation and cortical parcellation (aparc+aseg) were obtained from T1W and T2W images using FreeSurfer v7.1 recon-all command (Fischl, 2012), from which the binary tissue mask for the three regions of interest (ROIs), including the cerebral WM, the cerebral cortex, and the subcortical deep GM (cerebral GM minus the cerebral cortex), were derived. WMH were traced on the FLAIR image using ITK-SNAP software (version 3.8)^1^ (Yushkevich et al., 2006). To reduce partial volume effects at the tissue-CSF interface on regional CSFF measurements, more conservative tissue ROIs were generated for regional WF measurements by first applying a 1 mm isotropic erosion to the original FreeSurfer ROIs, followed by another erosion such that the distance between the ROI boundary and the boundaries of the ventricles and the subarachnoid space, i.e., CSF-filled intracranial spaces, was at least 1 mm in-plane and 5 mm through-plane (chosen based on the 1.3 × 1.3 × 5 mm^3^ voxel size of the FAST-T2 acquisition). To further study the contamination effect of ventricular and subarachnoid CSF on the cortical CSFF measurements, cortical masks were also generated without any erosion and with erosion such that the distance between the cortical tissue within the mask and the ventricular and subarachnoid CSF border was at least 1 mm in-plane and varying from 1 to 7 mm through-plane.

Since multi-exponential fitting is sensitive to changes in the input data, extra care was taken to minimize the signal bias in the FAST-T2 images. Gibbs ringing artifacts, predominantly in the slice direction due to the relatively thick 5 mm acquired slices, were mitigated using a local subvoxel-shifts Gibbs correction algorithm (Kellner et al., 2016) [*mrdegibbs* command in MRtrix3 software package (Tournier et al., 2019)]. Additionally, we applied correction for the noise bias inherent in the FAST-T2 sum-of-squares coil-combined magnitude data, which follows non-central chi distribution (Constantinides et al., 1997) and mainly affects the later low signal-to-noise ratio (SNR) echoes (the tail of the T2 signal decay curve). For this purpose, the number of the coil elements active during the FAST-T2 acquisition was determined from the DICOM image header, and the magnitude noise statistics were estimated from ROIs placed in the background air region of the two edge slices acquired at the last TE (these were chosen to minimize the effect of the anatomical signal for accurate noise estimation). The corrected FAST-T2 signal amplitude was then obtained from the measured noisy magnitude signal on a per-voxel basis by using a lookup table calculated for the given number of active coil elements following Eq. 2 in Constantinides et al. (1997).

Whole-brain MWF, IEWF and CSFF maps were computed from the Gibbs- and noise-corrected FAST-T2 magnitude image data using a multi-voxel three-pool non-linear least-squares fitting algorithm with a Laplacian local spatial smoothness constraint (Andrews et al., 2005; Nguyen et al., 2016). Following previous multi-exponential brain T2 mapping studies (MacKay et al., 1994; Whittall et al., 1997; Kolind et al., 2009; Prasloski et al., 2012), the effects of water exchange and compartmental T1 relaxation were not considered in our signal model. The lower and upper T2 bounds for each of the three water pools (in milliseconds) were set to [5 20], [20 200], and [200 2000] as in Nguyen et al. (2016). Following the conventional T2 relaxometry nomenclature (Lancaster et al., 2003; MacKay et al., 2006; Canales-Rodriguez et al., 2021), these water compartments represent short-T2 myelin water, intermediate-T2 intra/extra-cellular water (e.g., axonal and interstitial water), and long-T2 free and quasi-free water (mainly CSF) (MacKay et al., 2006), respectively. At each voxel, the T2 value of the myelin water and CSF components was initialized to 10 and 2,000 ms (Spijkerman et al., 2018), respectively, while the initial T2 value of the intra/extra-cellular water component was calculated using an efficient linear least-squares fit of the natural logarithm of the first four echoes (Otto et al., 2011). The amplitudes of the myelin water and intra/extra-cellular water components were initialized to be 10 and 90% of the signal measured at the first TE, respectively, while the amplitude of the CSF component was set to the signal measured at the last TE. A limited-memory Broyden-Fletcher-Goldfarb-Shanno (L-BFGS) algorithm implemented in MATLAB R2022a (The MathWorks Inc, Natick, MA, USA) was used to obtain the constrained non-linear least-squares solution, resulting in three compartmental water T2 values and three corresponding water signal amplitudes per image voxel (Schmidt et al., 2009). The relative MWF, IEWF, and CSFF maps were calculated as the ratios of the signal of the myelin water, intra/extra-cellular water, and CSF, respectively, to the total water signal within a voxel. The water maps were then aligned to the FreeSurfer space by linear rigid-body registration (six degrees of freedom) using the FLIRT algorithm (Jenkinson and Smith, 2001).

### 2.4. Statistical analysis

Statistical analysis was performed in the R environment (version 4.2.2; R Foundation for Statistical Computing, Vienna, Austria). Data are presented as mean ± SD. A *p*-value of less than 0.05 was considered statistically significant. Following visual inspection of the extracted mean MWF, IEWF, and CSFF data obtained in the cerebral deep GM, cerebral cortex, and cerebral WM as a function of age, we considered two multiple linear regression models with each of these regional WF measurements as an outcome variable (nine in total) and age as the main predictor variable of interest, adjusting for sex and ROI volume [uneroded and normalized to the subject skull size using the SIENAX algorithm (Smith et al., 2002) to account for the effect of brain atrophy in normal aging (Resnick et al., 2003; Raz et al., 2004)]:


$$\mathrm{Linear}\,\mathrm{model}\,\left(\mathrm{LM}\right):\text{WF}=\beta_{0}+\beta_{1}\,\text{Age}+\beta_{2}\,\text{Sex}+\beta_{3}\,\text{Volume}+\varepsilon$$

$$\mathrm{Quadratic}\,\mathrm{model}\,\left(\mathrm{QM}\right):\text{WF}=\beta_{0}+\beta_{1}\,{\text{Age}}^{2}+\beta_{2}\,\text{Age}+\beta_{3}\,\text{Sex}$$

$$+\beta_{4}\,\text{Volume}+\varepsilon$$

The final regression model was selected as the most parsimonious model that still fits the data adequately using an ANOVA to test if the squared term for age should be included in the model. If the difference was statistically significant, the QM was selected, otherwise the LM was selected. To correct for multiple comparisons, the *p*-values obtained for the age variable from the nine regression models were adjusted using the false discovery rate (FDR) method (Benjamini and Hochberg, 1995). Intra-scanner repeatability of the regional MWF, IEWF, and CSFF measurements was assessed using Bland-Altman plots (Bland and Altman, 1986). Finally, to assess the relationship between CSF spaces measured on a macroscopic (organ level) and microscopic (tissue level) scale, we calculated the Spearman’s rank correlation between the normalized lateral ventricle volume and regional CSFF measurements.

### 2.5. Availability of the data

Data are available upon reasonable request and a formal data sharing agreement between the authors’ and the requesting researchers’ institutions.

## 3. Results

**FIGURE 2:**
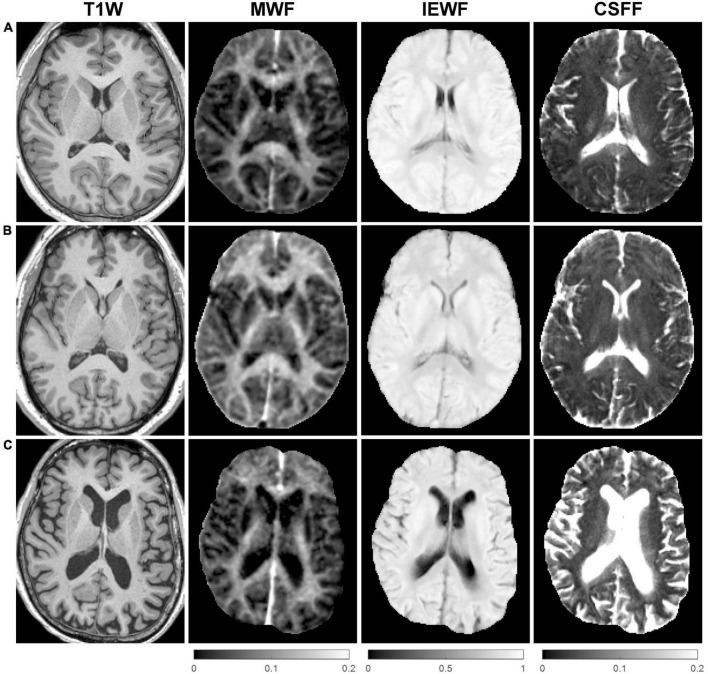
Representative axial mid-brain structural T1-weighted (T1W) magnetization-prepared rapid acquisition gradient echo (MPRAGE) images as well as corresponding myelin water fraction (MWF), intra/extra-cellular water fraction (IEWF), and cerebrospinal fluid fraction (CSFF) maps derived from fast acquisition with spiral trajectory and adiabatic T2prep (FAST-T2) data acquired from **(A)** a 22-year-old male, **(B)** a 46-year-old male, and **(C)** a 73-year-old female cognitively normal (CN) volunteers. Note the marked increase in cerebral atrophy, cortical loss, and lateral ventricular volume on the T1W image, and higher parenchymal CSFF values, in the latter subject compared to the younger subjects.

Figure 2 shows representative axial mid-brain WF maps obtained by the FAST-T2 sequence from 3 subjects. Compared to the younger 22-year-old (Figure 2A) and 46-year-old (Figure 2B) subjects, the 73-year-old subject (Figure 2C) showed prominent structural brain changes, including cerebral atrophy, cortical loss, and ventricular dilation seen on the T1W image, as well as increased values in the brain parenchyma on the CSFF map.

**FIGURE 3:**
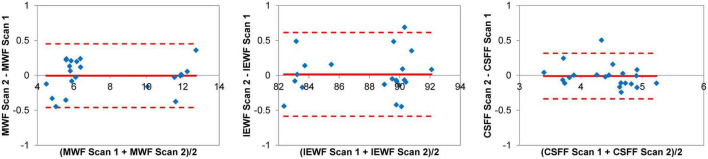
Bland-Altman plots of regional myelin water fraction (MWF), intra/extra-cellular water fraction (IEWF), and cerebrospinal fluid fraction (CSFF) measurements obtained by repeated fast acquisition with spiral trajectory and adiabatic T2prep (FAST-T2) scans in the cerebral white matter (WM), deep gray matter (GM) and cortex of seven cognitively normal (CN) volunteers, showing a negligible bias and narrow 95% limits of agreement of approximately ±0.5%.

The Bland-Altman plots of the regional MWF, IEWF, and CSFF values obtained in the cerebral WM, deep GM, and cortex from seven subjects (Figure 3) show that these measurements were highly repeatable with a negligible bias (–0.004% for MWF, 0.014% for IEWF, and –0.010% for CSFF) and a narrow 95% limits of agreement (approximately ±0.5%).

**FIGURE 4:**
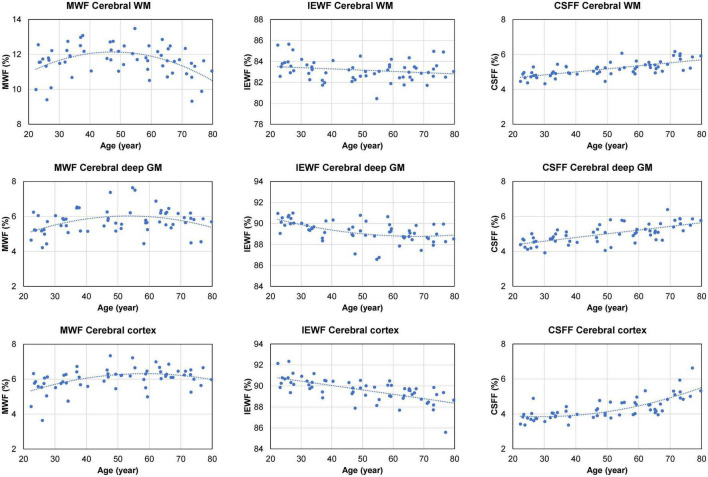
Scatter plots and trend lines (fitted using a second-order polynomial function) showing the cross-sectional mean myelin water fraction (MWF), intra/extra-cellular water fraction (IEWF), and cerebrospinal fluid fraction (CSFF) values versus age measured in the cerebral white matter (WM), cortex, and deep gray matter (GM) of 60 cognitively normal (CN) subjects.

Figure 4 shows the scatter plots and corresponding second-order polynomial trend lines of the cross-sectional MWF, IEWF, and CSFF values versus age obtained in the cerebral WM, cerebral cortex, and cerebral deep GM. MWF values exhibit an inverted U-shape in all three ROIs, while IEWF values decrease with age. The mean CSFF values in the cerebral cortex follow a quadratic trend and start to rise at approximately 50 years of age. However, in the cerebral WM and deep GM, CSFF values show a linear increasing trend with age. Multiple linear regression analysis with FDR correction revealed a statistically significant quadratic relationship between age and regional CSFF measurement (QM model) for the cerebral cortex (*p* = 0.018), after adjusting for sex and ROI volume. A statistically significant quadratic association with age was also found for MWF in the cerebral WM (*p* = 0.033), deep GM (*p* = 0.017), and cortex (*p* = 0.029), and for IEWF in the deep GM (*p* = 0.033). There was a statistically highly significant positive linear relationship between age and regional CSFF (LM model) in the cerebral WM and deep GM (*p* < 0.001). In addition, a statistically significant negative linear association between IEWF and age was found in the cerebral WM (*p* = 0.017) and cortex (*p* < 0.001).

**FIGURE 5:**
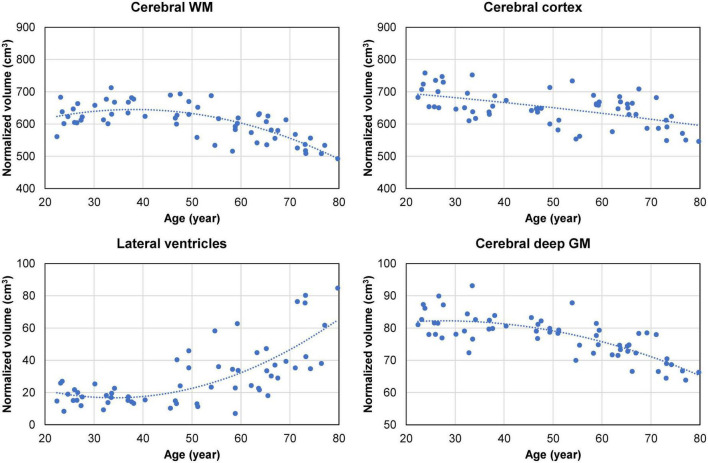
Cross-sectional brain volume data (normalized by the subject’s skull volume to account for difference in head size) obtained from 60 cognitively normal (CN) adults. The volume of the cerebral white matter (WM) and subcortical deep gray matter (GM) shows a quadratic decreasing trend starting in the middle age (approximately at age 40). This is accompanied by a quadratic increase in the volume of the lateral ventricles, with notable enlargement observed in some subjects in the 70–80 age group. The volume of the cerebral cortex exhibits a gradual linear decreasing trend with age.

Overall, the normalized cerebral WM and deep GM volumes exhibited a decreasing trend after the age of approximately 50 years, accompanied by an increase in the size of the lateral ventricles (Figure 5). The normalized volume of the cerebral cortex shows a linear decreasing trend with age (Figure 5). Spearman’s rank correlation analysis showed a statistically significant correlation between the normalized lateral ventricle volume and the regional CSFF measurement from the cerebral WM (ρ = 0.64, *p* < 0.001), cortex (ρ = 0.62, *p* < 0.001), and deep GM (ρ = 0.66, *p* < 0.001). The median WMH volume identified on the FLAIR image of subjects aged 45 and above (*n* = 37) was 0.13 cm^3^ (interquartile range, 0.02–0.56 cm^3^; range, 0–7.97 cm^3^).

**FIGURE 6:**
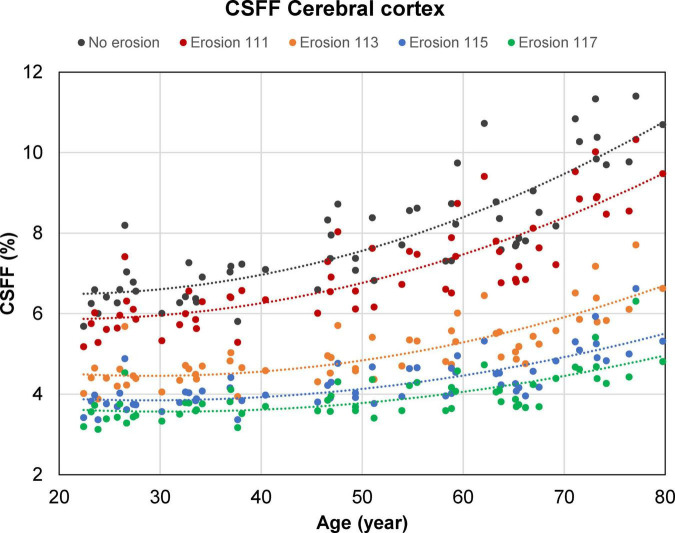
Scatter plots and trend lines (fitted using a second-order polynomial function) showing the cross-sectional mean cerebrospinal fluid fraction (CSFF) values versus age measured in the cerebral cortex of 60 cognitively normal (CN) subjects as a function of the cortical mask erosion. Starting with an uneroded cortical mask obtained from the FreeSurfer processing, erosion was applied in a progressive manner such that the distance between the cortical voxels within the eroded mask and the ventricular and subarachnoid cerebrospinal fluid (CSF) border is at least 1 mm in-plane and 1, 3, 5, or 7 mm through-plane (for example, “erosion 113” indicates erosion by 1 and 3 mm in the in-plane and through-plane direction, respectively).

Figure 6 shows the cortical CSFF values versus age as a function of the cortical mask erosion. Note that as the mask erosion is increased and consequently, fewer cortical voxels are affected by the contamination from the global CSF in the subarachnoid and ventricular spaces, the cortical CSFF versus age curves trend downward but maintain the quadratic relationship with age.

## 4. Discussion

In this cross-sectional MRI study of age-related changes in compartmental brain tissue water of CN subjects aged 20–80 years, we found strong evidence that parenchymal CSFF, a potential biomarker of the CSF space within a brain voxel, is associated with age. After adjusting for sex and brain ROI volume (to account for cerebral atrophy), we found a statistically significant quadratic relationship between age and regional CSFF in the cerebral cortex. To the best of our knowledge, this finding has not been reported in the literature and may provide new insights into reorganization of brain tissue water and its association with CSF-driven glymphatic clearance. In addition, we found a statistically significant positive linear association between age and CSFF in the cerebral WM and deep GM. This is contrasted with the age-related change of the intra/extra-cellular water compartment, as measured by IEWF, that decreases linearly with age in the cerebral WM and cortex, and quadratically in the deep GM. The MWF follows an inverted U-shape with a statistically significant quadratic relationship with age in all three ROIs (cerebral WM, cortex, and deep GM). The different temporal patterns of the cross-sectional CSFF, IEWF and MWF change with age (Figure 4) likely reflect biological and physiological changes, including, but not limited to, neuronal atrophy, breakdown and ballooning of myelin sheath, vascular changes, and altered fluid dynamics, in an aging brain (Liu et al., 2017; Banks et al., 2021). Further studies are warranted to elucidate the origin of the observed WF changes and their roles in the pathogenesis and progression of age-related neurodegenerative diseases.

Among the three brain tissue water compartments studied in this work, the CSF component is often regarded as a confounder in brain MRI techniques, including functional MRI and proton MR spectroscopic studies (Horska et al., 2002), and has not been studied extensively in the context of aging. Qin et al. performed single-compartment mapping of parenchymal CSF volume fraction (V_CSF_) using a T2-prepared 3D GRASE sequence in six healthy volunteers aged 32–50 years (Qin, 2011). They proposed to isolate the tissue CSF signal (T2∼2 s) from the signal of the other tissue water compartments (T2 < 120 ms) by acquiring multi-echo image data at long TEs > 600 ms. The data was fitted with a mono-exponential function to obtain the CSF signal at zero TE, which was then normalized by the ventricular pure CSF signal to obtain a V_CSF_ map. They reported mean V_CSF_ of 8.9, 11.4, and 21.4% in the occipital, temporal, and frontal cortex, respectively, and V_CSF_ of 0–3% in WM voxels. Assuming brain GM and WM water content of 83 and 70% (Whittall et al., 1997), respectively, and cortical CSFF of 4.0% and WM CSFF of 5.0% for the 30–50 years age group (Figure 4), we obtained a V_CSF_ of 3.3 and 3.5% in the cerebral cortex and WM, respectively. The much higher cortical V_CSF_ values reported by Qin et al. are most likely due to the larger partial volume effect in their image data (voxel size = 53.7 versus 7.8 mm^3^ in our study). Also, a conservative tissue mask was used in our ROI analysis (the distance between ROI voxels and CSF-filled brain reservoirs, including the ventricles and the subarachnoid space, is at least 1 mm in-plane and 5 mm through-plane), which further reduces partial voluming with the macroscopic CSF signal. In a more recent study, Canales-Rodriguez et al. (2021) applied a voxel-based analysis to three-compartment T2 relaxometry data obtained using a 3D GRASE sequence (Prasloski et al., 2012) in 145 healthy subjects aged 18–60 years. They found that parenchymal CSFF (termed “free and quasi-free water fraction” in their work) generally increases linearly with age, with a much larger slope observed in men than in women. Their reported brain CSFF values, ranging approximately 5–18%, are also higher than our results, which is likely due to the increased partial volume effect (voxel size = 42.9 versus 7.8 mm^3^ in our study) and differences in the data acquisition and post-processing methods. An important extension of the study in Canales-Rodriguez et al. (2021) is the inclusion of CN subjects in the 60–80 years age group and it is precisely in this group that a strong age effect on the cortical CSFF is demonstrated.

In this study, we observed an inverted U-shape in the age-dependent MWF change in the cerebral WM, deep GM, and cortex (Figure 4). A statistically significant quadratic relationship with age was found in all three ROIs. While MWF is an established quantitative marker of myelin loss in demyelinating diseases such as MS (Laule et al., 2008; Mancini et al., 2020; van der Weijden et al., 2021), previous investigations in healthy individuals have reported inconsistent MWF trends with age, which could increase (Flynn et al., 2003; Lang et al., 2014; Canales-Rodriguez et al., 2021) or decrease (Faizy et al., 2018) linearly, remain stable (Billiet et al., 2015), or follow a quadratic inverted U-shape (Arshad et al., 2016; Papadaki et al., 2019; Bouhrara et al., 2020; Dvorak et al., 2021). A possible explanation for this discrepancy could be the difference in the age range of the study cohorts. As age-related changes in myelin sheath, axons, and myelin-producing oligodendrocytes in humans are complex and not well-understood (Liu et al., 2017), further validations of the specificity of MWF and other myelin biomarkers in normal aging are needed.

The rise in parenchymal CSFF in the cerebral cortex after the age of 50 (Figure 4) is accompanied by the enlargement of the lateral ventricles (Figure 5), posing the question whether these two phenomena are related. Since the dilation of the lateral ventricles is a measure of cerebral atrophy (Nestor et al., 2008), we adjusted for the effect of age-dependent cerebral ROI volume on CSFF in the multiple linear regression model and found that age remained a statistically significant variable. We also found that the correlation between the lateral ventricle size and the regional CSFF measurements was statistically significant but only moderate (Spearman’s correlation < 0.7). The correlation was notably poor for subjects in the 70–79 years age group, with about half of the group having much larger lateral ventricles (Figure 5) yet similar regional CSFF values compared to the other half. These results suggest that the macroscopic (organ level) and microscopic (tissue level) CSF spaces are related, but CSFF is a tissue marker independent from the observable macroscopic brain atrophy and ventricular dilation. Another potential explanation for the observed higher CSFF in the WM is the frequently observed WMH in older subjects (Habes et al., 2018; Moura et al., 2019; Garnier-Crussard et al., 2020). However, the WMH volume in our study cohort (median 0.13 cm^3^) was much smaller than the WM volume (median 458 cm^3^). Therefore, excluding WMH from the cerebral WM ROI has negligible effects on the regional measurements and consequently on the statistical results (data not shown). A possible interpretation of CSFF increasing with age is the dilation of the microscopic PVS due to glymphatic fluid stasis caused by impaired glymphatic clearance of metabolic wastes in the aging brain (Nedergaard, 2013; Wardlaw et al., 2020). PVS enlargement is conventionally detected and quantified on T2W images (Ramirez et al., 2016; Ballerini et al., 2018). A recent cross-sectional study of 1789 healthy subjects aged 8–100 years revealed a biphasic PVS volume change with age in the basal ganglia and the WM, slightly decreasing until the mid-20 s and increasing afterward (Kim et al., 2023); this is similar to the trends seen in our study (Figure 4). However, unlike CSFF, there was a considerable variation in PVS volume fraction, especially in the older subjects (Kim et al., 2023). Since this approach is not capable of detecting PVS much smaller than the voxel size (approximately 1 mm), future work is needed to study the association between the T2W-visible PVS load and CSFF in the same cohort.

Several studies have reported a decreasing trend with age of IEWF in various brain regions (Billiet et al., 2015; Canales-Rodriguez et al., 2021) in agreement with our findings. Between the age of 20 and 80 years, we found the reduction in IEWF is about 0.7% in WM and 1.6% in GM (in absolute terms), which is accompanied by a similar increase in CSFF. Since the intra/extra-cellular water compartment is the dominant source of water in the brain tissue (IEWF ∼85% in WM and ∼90% in GM and), a decrease in this water compartment alone does not fully explain a corresponding increase in the tissue CSF water. As an illustrative example, assume the water content of GM is 1 g/mL, which consists of 0.05 g/mL myelin water, 0.90 g/mL intra/extra-cellular water, and 0.05 g/mL CSF. A loss of water in the intra/extra-cellular compartment alone (from 0.90 to 0.76 g/mL) will result in a 1.6% decrease in IEWF, but only a 0.8% increase in CSFF. Therefore, the observed CSFF increase in the GM indicates that there is an increase in the CSF compartment. To ascertain the true magnitude of change in these water compartments, a voxel-wise mapping of the brain tissue total water content (Neeb et al., 2006; Meyers et al., 2017; Nguyen et al., 2017) is needed and will be considered in our future work. Knowledge of the changes in the absolute compartmental water content is also essential for elucidating the biological origin of the observed IEWF decrease, which is beyond the scope of the current work.

The present study has several limitations. First, our sample size is small, which restricts the number of ROIs upon which inference can be performed in the statistical analysis. Second, due to scan time constraints, we did not measure the tissue water content, which makes the interpretation of the changes in the compartmental WF, a relative measure, somewhat challenging. Third, current multi-component T2 relaxometry techniques cannot differentiate intra-cellular and interstitial water compartments within a voxel. In our future work, we will consider combining FAST-T2 with diffusion imaging approaches such as neurite orientation dispersion and density imaging (NODDI) (Zhang et al., 2012) and free-water DTI (Pasternak et al., 2009) which are capable of separating these water compartments. Fourth, the FAST-T2 imaging slice is thick (5 mm), which necessitates an aggressive ROI mask erosion in the slice (inferior-superior) direction to mitigate the partial volume effects with CSF in the ventricular and subarachnoid spaces. However, partial volume effects at the GW and WM interface may exist. Fifth, due to the retrospective nature of our study, there was a race imbalance in our cohort. Although including race as a covariate in the multiple regression models does not change our statistical results (data not shown), more studies are needed to confirm these findings. Sixth, since our study subjects were imaged with two different T2W sequences with different image resolution and contrast, and also because the CSFF maps were acquired in much thicker 5 mm slices, we did not consider CSFF measurements in PVS identified on T2W images. Finally, we made the assumption that CSFF mainly captures the contribution from CSF water, which is reasonable based on the current understanding of the water T2 spectrum in the healthy brain tissue (MacKay et al., 2006). It should be noted that that it is difficult to achieve accurate T2 estimation of this long-T2 water component given that our last TE is only ∼300 ms. A previous brain water mapping study using 48 TEs up to 1,120 ms reported a pathological water compartment in the 200–800 ms range in multiple sclerosis lesions (Laule et al., 2007). Therefore, further independent validations, by histology or other imaging methods, are required to confirm our findings.

In conclusion, brain tissue water in different compartments shows complex age-dependent patterns across the adult lifespan of 20–80 years. Parenchymal CSFF, a measure of subvoxel CSF-like water in the brain tissue, is quadratically associated with age in the cerebral cortex and linearly associated with age in the cerebral WM and deep GM.

## Data availability statement

The raw data supporting the conclusions of this article will be made available by the authors, without undue reservation.

## Ethics statement

The studies involving human participants were reviewed and approved by IRB at Weill Cornell Medicine. The patients/participants provided their written informed consent to participate in this study.

## Author contributions

YL and TN: conceptualization. TN: MRI sequence development. YL, ML, AK, YW, TN, SG, GC, and JI: study design. LZ, TN, and XW: image processing. ES, LZ, and TN: data analysis. LZ, TN, and YL: original draft. LZ, YL, ES, XW, AK, GC, JI, YW, SG, ML, and TN: review and editing. All authors contributed to the article and approved the submitted version.

## Footnotes

1
http://www.itksnap.org
